# Human ophthalmic artery as a sensor for non-invasive intracranial pressure monitoring: numerical modeling and in vivo pilot study

**DOI:** 10.1038/s41598-021-83777-x

**Published:** 2021-02-26

**Authors:** Paulius Lucinskas, Mantas Deimantavicius, Laimonas Bartusis, Rolandas Zakelis, Edgaras Misiulis, Algis Dziugys, Yasin Hamarat

**Affiliations:** 1 Health Telematics Science Institute, Kaunas University of Technology, K. Barsausko Str. 59-A556, 51423 Kaunas, Lithuania; 2 Laboratory of Combustion Processes, Lithuanian Energy Institute, Breslaujos Str. 3, 44403 Kaunas, Lithuania

**Keywords:** Neurology, Medical research

## Abstract

Intracranial pressure (ICP) monitoring is important in managing neurosurgical, neurological, and ophthalmological patients with open-angle glaucoma. Non-invasive two-depth transcranial Doppler (TCD) technique is used in a novel method for ICP snapshot measurement that has been previously investigated prospectively, and the results showed clinically acceptable accuracy and precision. The aim of this study was to investigate possibility of using the ophthalmic artery (OA) as a pressure sensor for continuous ICP monitoring. First, numerical modeling was done to investigate the possibility, and then a pilot clinical study was conducted to compare two-depth TCD-based non-invasive ICP monitoring data with readings from an invasive Codman ICP microsensor from patients with severe traumatic brain injury. The numerical modeling showed that the systematic error of non-invasive ICP monitoring was < 1.0 mmHg after eliminating the intraorbital and blood pressure gradient. In a clinical study, a total of 1928 paired data points were collected, and the extreme data points of measured differences between invasive and non-invasive ICP were − 3.94 and 4.68 mmHg (95% CI − 2.55 to 2.72). The total mean and SD were 0.086 ± 1.34 mmHg, and the correlation coefficient was 0.94. The results show that the OA can be used as a linear natural pressure sensor and that it could potentially be possible to monitor the ICP for up to 1 h without recalibration.

## Introduction

Continuous intracranial pressure (ICP) monitoring is limited due to the invasive nature of some techniques. Nevertheless, the high risk/benefit ratio allows for the usage of invasive ICP monitors in certain groups of patients who could have sudden pathophysiological ICP variation. Such spontaneous ICP changes are common in traumatic brain injury (TBI) patients^1^. Therefore, invasive ICP monitoring is mostly performed in patients with severe TBI (Glasgow Coma Scale score of 3–8)^2^. The gold-standard ICP-monitoring method is considered to be an intraventricular drain connected to an external pressure transducer. Another major technique for continuous ICP monitoring involves intraparenchymal devices, which are not as accurate as intraventricular drains and cannot be recalibrated once inserted^3,4^.

ICP monitoring is in demand in many clinical applications, including conscious patients (ophthalmology, neurology, aerospace medicine, etc.). In the case of ophthalmology, glaucoma is the second leading cause of irreversible vision loss worldwide. It affected 76 million people in 2020 and is predicted to reach 111.8 million cases by 2040^5^. Prospective and retrospective studies using lumbar puncture technique have revealed that glaucoma patients have lower ICP than age-matched healthy subjects^6–9^, which suggests that ICP might be one of the influential factors in the pathophysiology of glaucoma^10^. However, ICP monitoring is not employed in daily practice for glaucoma patients due to its invasiveness. In the case of aerospace medicine, visual impairment of astronauts who are exposed to long-term microgravity is induced by changes in ICP^11^, which must be monitored non-invasively in this case. Unfortunately, such a non-invasive device is not available in clinical practice.

Ragauskas et al. reported a novel non-invasive snapshot method for the measurement of ICP based on the two-depth transcranial Doppler (TCD) technique, which can be used for simultaneously measuring blood flow velocities in the intracranial and extracranial segments of the ophthalmic artery (OA)^12^. In this method, the intracranial segment of the ophthalmic artery (IOA) is compressed by ICP while the extracranial segment of the ophthalmic artery (EOA) is compressed by externally applied pressure (Pe). Blood flow velocity parameters in both of these OA segments are approximately equal when Pe = ICP. A review paper has discussed many TCD-based methodological approaches for non-invasive ICP monitoring, and the overall accuracy is around ± 12 mmHg^13^. The innovative two-depth TCD-based non-invasive ICP snapshot measurement technique has clinically acceptable accuracy, precision, and diagnostic reliability^14–16^.

The aim of this study was to investigate the possibility of using the OA as a pressure sensor for continuous ICP monitoring. The idea is that periodic non-invasive ICP snapshot measurements^12^ could be used for non-invasive recalibration of a non-invasive ICP monitoring method. A key problem in glaucoma diagnosis and treatment is minimizing the deformation of the lamina cribrosa caused by the pressure difference between the intraocular pressure (IOP) and ICP (IOP-ICP)^17^. Our previous studies showed that our non-invasive ICP snapshot measurement method is accurate and precise enough to be applied for abnormally low ICP value diagnosis in normal-tension glaucoma (NTH) and high-tension glaucoma (HTG) patients^10,18^.

ICP and IOP have circadian fluctuations over time, so snapshot measurements of these pressures at a certain time do not provide sufficient diagnostic information^19,20^. The monitoring of ICP and IOP is needed for precise and individual patient specific NTG and HTG diagnosis in cases where ICP values are below the normal range. In this study, numerical modeling was employed to investigate the possibility of using OA as an ICP sensor for monitoring. Then, a pilot clinical study was conducted to obtain the first clinical evidence on the linearity and accuracy of the OA as an ICP sensor.

## Methods

### Numerical modeling of ophthalmic artery as a pressure sensor

**Figure 1:**
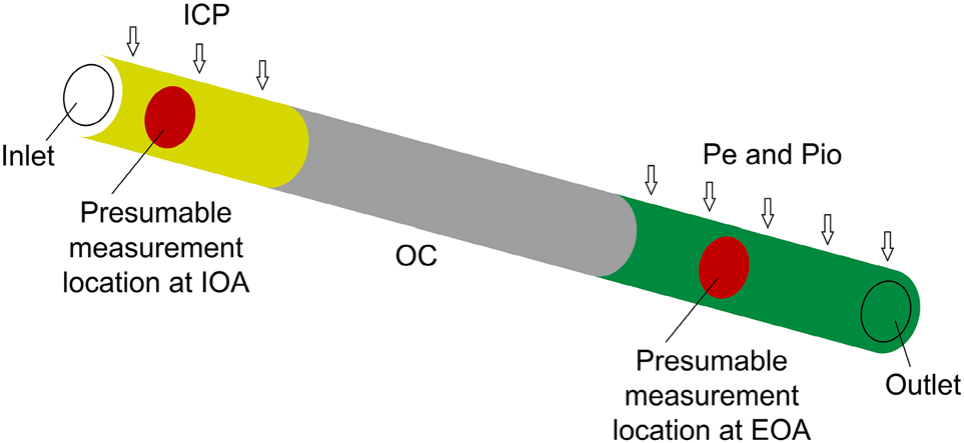
Diagram of the numerical model of an idealized straight ophthalmic artery. *ICP* intracranial pressure, *Pe* externally applied pressure, *Pio* the intraorbital pressure, *IOA* intracranial segment of the ophthalmic artery, *EOA* extracranial segment of the ophthalmic artery, *OC* optic canal.

To understand and prove the concept of the non-invasive ICP monitoring method, it is important to estimate how the dynamics of the blood flow in the OA is affected during the monitoring procedure. Therefore, numerical modeling was performed on a straight idealized OA (Fig. 1).

COMSOL Multiphysics software (v.5.1 COMSOL AB, Stockholm, Sweden) was used to solve the fluid–structure interaction (FSI) model of a compliant OA while considering the two-way coupling between the pulsatile blood flow and the artery wall (please see Online Appendix 1 for more details).

The following values of acting pressures were considered and used only for numerical modeling of non-invasive ICP snapshot measurements: ICP = {0, 10, 20, 30} mmHg^21^, intraorbital pressure Pio = 4 mmHg^22^, and Pe = [0:2:34] mmHg^12,23,24^.

For every combination of the considered parameters, three cardiac cycles were modeled. During the initial cardiac cycle, all the acting pressures were ramped up from zero to the prescribed values, while during the second cardiac cycle the momentum produced by the initial ramp-up settled down. The difference of the temporal results of the cross-sectional areas at presumable measurement locations of OA between the second and third heart pulse cycle differed by less than 0.01% over the complete cardiac cycle. Therefore, it was concluded that the momentum produced by the initial ramp had negligible influence on the results of the third heart pulse cycle, and the results of the third cardiac cycle were used for the analysis by collecting data at every 0.004 s. This resulted in a 250 time moments per cardiac cycle.

### Blood flow factor used for the non-invasive ICP monitoring

The cross-sectional areas in the intracranial and the extracranial segments of the OA depend on the pressures acting on the walls of the OA. Furthermore, the cross-section area of the artery is associated with the intensity of the Doppler signal (Online Appendix 2). Consequently, variations of the OA’s cross-sectional area in the intracranial and extracranial segments can be related to ICP by the integral factors derived from the intensity of the Doppler signal. We constructed the blood flow factor ${\text{BFF}}$ for non-invasive ICP monitoring as an integral of the logarithm of the ratio of the Doppler signal intensity *P*_*s*_ obtained from the IOA and the EOA segments of the OA (Online Appendix 2).

### Clinical material

The non-invasive ICP (ICP_non-inv_) monitoring method was investigated clinically after numerical modeling. This pilot study was conducted at the Department of Neurosurgery, Republic Vilnius University Hospital. The study was based on 7 TBI patients that underwent surgery, during which ICP monitoring sensors were implanted. The exclusion criteria were age less than 18 years and a lack of invasive ICP or arterial blood pressure (ABP) data.

The study was approved by the Vilnius Regional Biomedical Research Ethics Committee (No. 158200-15-801-323, date: 2015-10-06), and parents/legal representative of participants provided written informed consent, according to the Declaration of Helsinki. ICP monitoring data were collected between May 2016 and January 2018.

### ICP monitoring and data sampling

**Figure 2:**
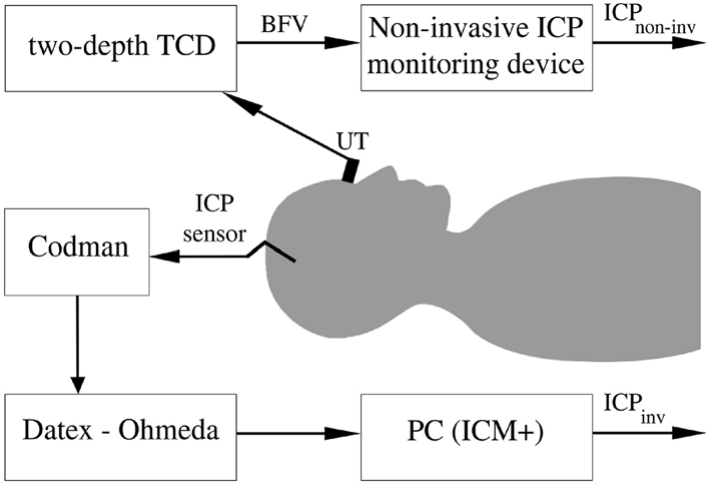
The set-up for simultaneous invasive and non-invasive intracranial pressure monitoring. *UT* ultrasonic transducer, *BFV* blood flow velocity, *TCD* transcranial Doppler, *ICP*_*inv*_ invasively monitored intracranial pressure, *ICP*_*non-inv*_ non-invasively monitored intracranial pressure.

The simultaneous invasive and non-invasive ICP monitoring is illustrated in Fig. 2. Invasive ICP was measured by a Codman ICP monitor with a catheter tip sensor (Johnson & Johnson Professional, Inc., Raynham, MA, USA). It was sampled at 300 Hz and recorded on a computer by ICM+ software (version 8.2; ICM+ software, University of Cambridge, UK) with a vital sign monitor (Datex-Ohmeda, Inc., Madison, WI, USA).

In the case of non-invasive ICP monitoring, a 2-MHz ultrasonic transducer was mounted on an individually fitted head frame. The locations of the IOA and EOA segments were determined using the edge of the internal carotid artery as a reference point^25^. The blood flow velocities of both segments of the OA were recorded using a two-depth TCD. Signal processing was performed to remove artifacts and to calculate BFF data points with a sampling frequency of 0.1 Hz. In this pilot study, individual invasive ICP readings were used for initial patient specific BFF data calibration to obtain non-invasive ICP values in pressure units. Next, we monitored ICP continuously for a 1 h period without recalibration. In contrast to the non-invasive ICP snapshot measurement method, Pe was not needed for the non-invasive ICP monitoring procedure.

### Statistics

Typically, one paired set of ICP_inv_ and ICP_non-inv_ data points was obtained every 10 s, resulting in a total of 360 data points during a 1 h monitoring session.

Paired data were analyzed using a Bland–Altman analysis. Linear regression analysis was used to test the hypothesis that the OA can be used as a linear ICP sensor.

MATLAB software (version R2015b; MathWorks Corporation, Natick, MA, USA) was used to process the data and to perform the Bland–Altman and linear regression analyses.

## Results

### Numerical modeling

**Figure 3:**
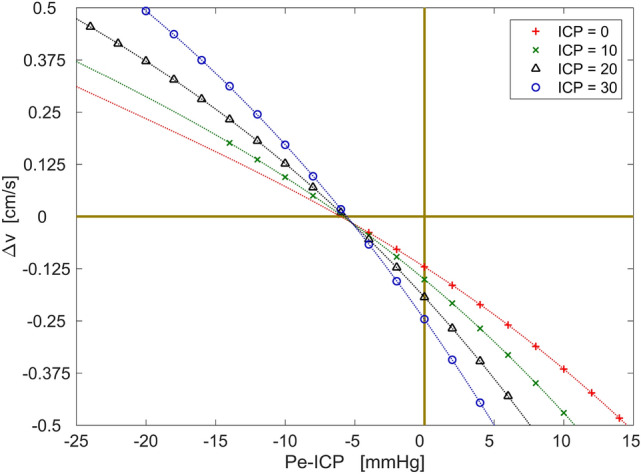
The difference of the blood flow velocities between presumed ICP_non-inv_ locations in the intracranial and extracranial segments of the ophthalmic artery with respect to the intracranial and extracranial pressures. The polynomial curves are fitted to the data points.

The blood flow velocities were extracted at the presumed ICP_non-inv_ monitoring locations in the IOA and EOA segments (Table 7 in Appendix 1). The obtained blood flow velocities were averaged across the artery lumen cross-sections in their vicinity and over the cardiac cycle. The difference between the resulting velocities in IOA and EOA segments is noted as ${\Delta v}$, which is close to zero when Pe is about 6 mmHg lower than the ICP (Fig. 3). This suggests that the systematic error of the physical ICP_non-inv_ monitoring is about 6 mmHg.

Part of this error is due to the intraorbital pressure, which was Pio = 4 mmHg in our case. Another part is due to the ophthalmic arterial pressure gradient, which was 1.25 mmHg. Such systematic errors are known a priori, so they can be eliminated in a software solution provided with the proposed non-invasive ICP monitoring technology. Consequently, the patient-specific component produced systematic error less than 1.0 mmHg. The patient-specific component incorporated the arterial pulse wave and the patient-specific mechanical non-equivalence between OA segments, which are not known a priori.

### Clinical study

Demographic data and clinical conditions of the patients are presented in Table 1. An individual linear calibration equation (Table 2) was used for each patient to calibrate BFF data points to derive non-invasive ICP values in pressure units. Then, the obtained values were compared with simultaneously monitored ICP_inv_. One out of the 7 patients was excluded due to invasive ICP or ABP not being monitored. TCD signal artifacts were then removed, after which a total of 1928 paired data points were obtained for the final comparison. The corresponding individual numbers of paired data points are presented in Table 2. The total mean and SD of the measured differences between ICP_inv_ and ICP_non-inv_ are 0.086 ± 1.34 mmHg. The individual means and SD are presented in Table 2.

**Table 1:** Demographic data and clinical conditions of the patients included in this pilot study.

Pat. no	Age, years	Gender	GCS on admission	Diagnosis	Surgery type	GOS
1	44	Male	3	DAI with skull open fractures	Intraparenchymal ICP	1
2	39	Male	8	DAI, SDH	Intraparenchymal ICP	5
3	26	Female	7	DAI, SDH	Intraparenchymal ICP	1
4	29	Female	5	DAI	Intraparenchymal ICP	4
5	20	Female	4	DAI	Intraparenchymal ICP	3
6	20	Male	7	DAI	Intraparenchymal ICP	3

*GCS* Glasgow coma scale, *GOS* Glasgow outcome scale, *DAI* diffuse axonal injury, *SDH* subdural hematoma, *ICP* intracranial pressure.

**Table 2:** The individual parameters obtained from each patient included in this pilot study.

Pat. no	Range of ICP_inv_, min–max, mmHg	Calibration equation used to calculate ICP_non-inv_	Measured differences, mean ± SD mmHg	Number of paired data points
1	12–22	56.3 × $\overline{BFF}$ + 10.1	0.43 ± 0.91	223
2	16–19	263.8 × $\overline{BFF}$ + 11.8	− 0.22 ± 1.23	360
3	8–11	70.4 × $\overline{BFF}$ + 6.2	− 0.65 ± 1.07	356
4	10–19	218.9 × $\overline{BFF}$ + 4.5	1.61 ± 1.61	280
5	8–10	109.8 × $\overline{BFF}$ + 3.8	0.18 ± 0.64	360
6	13–17	586.2 × $\overline{BFF}$ + 6.9	− 0.39 ± 1.20	349

**Figure 4:**
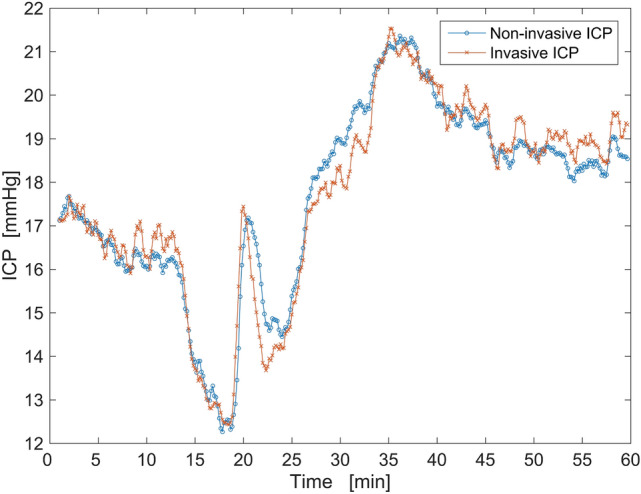
An example of paired invasive and non-invasive ICP data points of the first patient after filtering (60-s moving average filter).

**Figure 5:**
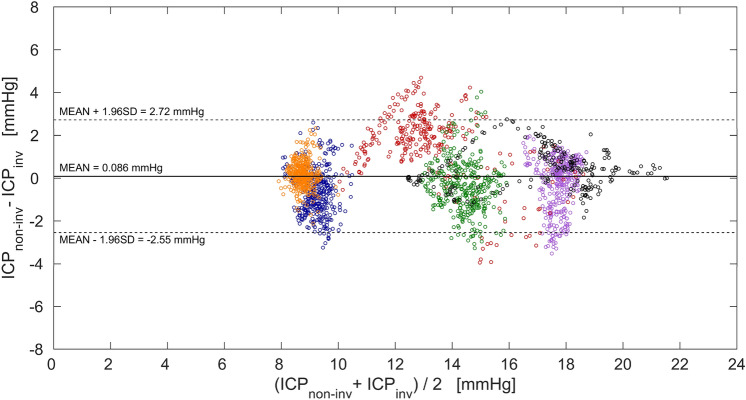
Bland–Altman plot showing the distribution of the measured differences between intracranial pressure monitored directly using invasive Codman ICP monitor (ICP_inv_) and indirectly using two-depth transcranial Doppler (ICP_non-inv_) in the entire pressure range obtained in all six patients. Paired data points collected from each of the six patients are separated by colors: first patient—black, second patient—violet, third patient—blue, fourth patient—red, fifth patient—orange, sixth patient—green. The thick horizontal line indicates the total mean of the measured differences, while the two dashed horizontal lines indicate the standard deviation (SD) of ± 1.96 of the measured differences.

As an example, Fig. 4 shows a plot of 223 paired invasive and non-invasive ICP monitoring data points of the first patient. A 60-s moving average filter was used for smoothing both readings. The maximum difference between the paired data points was 1.61 mmHg. The measured differences between ICP_inv_ and ICP_non-inv_ of all 6 patients are presented in Fig. 5 as a Bland–Altman plot. The extremes of the measured differences between ICP_inv_ and ICP_non-inv_ are − 3.94 and 4.68 mmHg, and 95% of the observations fall in the interval of − 2.55 to 2.72 mmHg.

**Figure 6:**
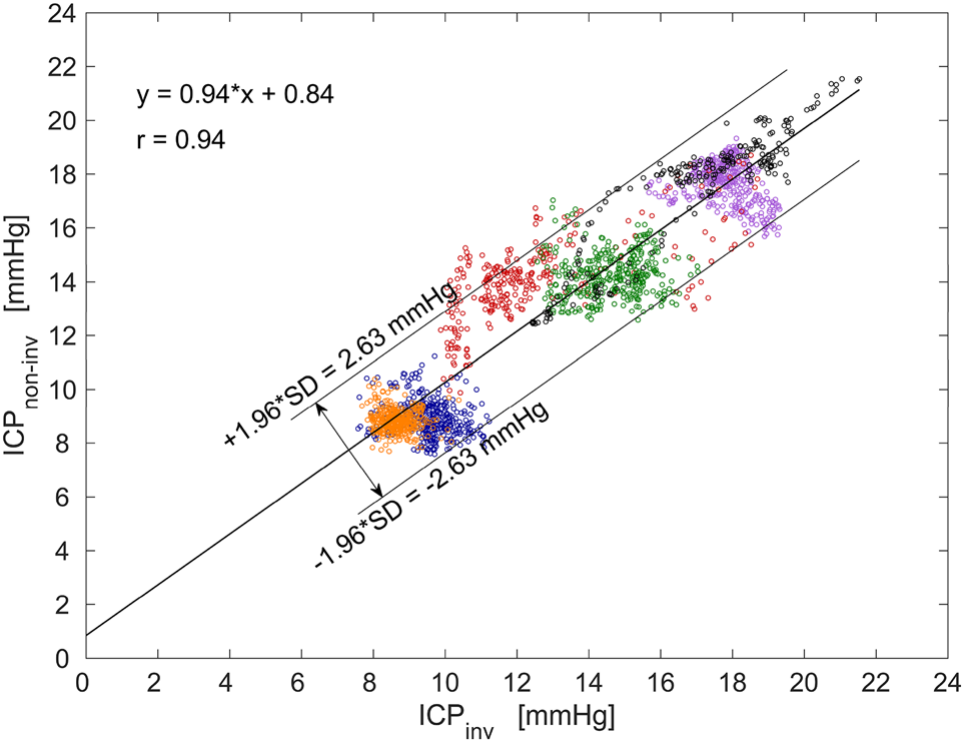
A linear regression graph of all data points shows a strong positive relationship (r = 0.94) between the non-invasively obtained intracranial pressure values (ICP_non-inv_) and the invasive ICP values (ICP_inv_). Paired data points collected from each of the six patients are separated by colors: first patient—black, second patient—violet, third patient—blue, fourth patient—red, fifth patient – orange, sixth patient—green.

The regression analysis showed that there is a strong positive relationship (r = 0.94) between the data obtained with two-depth TCD and the Codman ICP monitor (Fig. 6). The linear equation y = 0.94x + 0.84 demonstrates that OA can be used as a linear pressure sensor with a bias (systematic error) of 0.84 mmHg in the tested ICP range.

## Discussion

ICP monitoring has been used for decades in the management of TBI patients^26^. However, the demand for ICP monitoring is also increasing in the fields of ophthalmology^6,8,9,18^ and aerospace medicine^11,27^. In this study, we investigated the possibility of using the OA as a pressure sensor for non-invasive ICP monitoring based on two-depth TCD.

The modeling was performed to investigate the blood flow dynamics in the OA and the possible systematic error due to several mechanisms: the ophthalmic arterial pressure gradient, intraorbital pressure, arterial pulse wave, and patient-specific mechanical non-equivalence between IOA and EOA segments. The results of the modeling showed that errors of 4 mmHg and 1.25 mmHg were produced by the intraorbital pressure and the average ophthalmic arterial blood pressure gradient, respectively. The average ophthalmic arterial blood pressure gradient corresponds well with the theoretical value of 1.225 mmHg based on the Hagen–Poiseuille flow assumptions. The patient-specific component, resulting from the arterial pulse wave and the mechanical non-equivalence produced an error less than 1.0 mmHg. This error is unknown a priori, so it sets possible limiting boundaries for the random error. Based on the clinical standards the random error should be close to ± 2 mmHg^28^.

Previous studies showed that the systematic error of the modeled ICP_non-inv_ monitoring based on the blood velocity was within the limits of [1.6, 1.8] mmHg for the idealized straight OA and [0.04, 4.6] mmHg for the subject-specific curved OA^29,30^, which are similar to the value of ± 1.0 mmHg obtained in this study. The current state of the neuroimaging techniques allows for the implementation of subject-specific models considering the OA branching from the internal carotid artery. This allows for the quantitative estimation of the ICP_non-inv_ monitoring process and more detailed reasoning about the systematic error in specific patients.

In the pilot study, the non-invasive ICP monitoring method showed a strong positive correlation (r = 0.94) with invasively monitored ICP data. In order to test our hypothesis about the linearity of the OA results, invasive ICP values were used for initial calibration, which means the non-invasive two-depth TCD data is converted into non-invasive ICP values in pressure units. The regression analysis showed that the OA can act as a linear ICP sensor in the tested ICP range.

Several important limitations of our study must be mentioned. The main limitation of this pilot study is the small number of patients with relatively narrow ICP variation (8–22 mmHg). A substantially larger sample of patients with different conditions would guarantee the achievement of statistical significance in a prospective study. At the same time, the OA would be tested as a pressure sensor in a wide range of ICP values. Next, the reproducibility of the proposed method was not investigated in this study, although it has been tested by removing and re-applying the head frame in an ICP snapshot measurement study^14^. Both non-invasive ICP monitoring and snapshot measurement methods employ the same principles, which involve locating OA segments and using two-depth TCD data acquisition.

The accuracy of the ventricular ICP monitor depends on periodical recalibration by opening the transducer to atmospheric pressure and returning it to a zero reference point^31^. The patient positioning in relation to the transducer affects the accuracy of ICP readings^32^. The complications in the case of ventricular catheters included a 1–10% risk of infection and a 1–2% risk of bleeding^33^. Parenchymal monitoring devices also require calibration and zeroing, and the pneumatic ICP monitor even runs recalibration every 15 min during monitoring. The risk of complication in the case of parenchymal monitoring is lower compared to ventricular catheters, but it still exists nonetheless^33^.

The suggested method can monitor ICP non-invasively for up to one hour without recalibration. We used invasive ICP sensor data for initial calibration in this study, but a non-invasive ICP snapshot measurement method that has clinically acceptable accuracy and precision^14,16^ could be used for the initial calibration and periodic recalibration. In this case, the suggested method would be completely non-invasive and avoid any complications caused by invasive procedures.

## Conclusion

The numerical modeling and pilot clinical study have demonstrated that the OA can be used as a pressure sensor for non-invasive ICP monitoring. We have observed that it is possible to monitor ICP non-invasively for a one hour without recalibration. This technology could solve the problems of ICP monitoring and diagnosis in conscious subjects, including open-angle glaucoma patients. Nevertheless, further studies are needed to investigate the method in patients with relatively low and high ICP values.

## Figures and Tables

**Figure 7:**
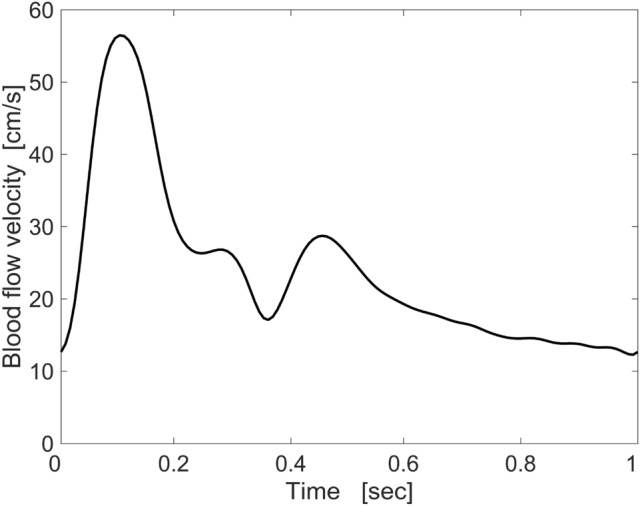
Typical maximum blood flow velocity waveform in ophthalmic artery that was implemented in the numerical model.

## Appendix 1: Numerical modeling of ophthalmic artery as a pressure sensor

The non-invasive intracranial pressure (ICP_non-inv_) monitoring was investigated numerically while considering an idealized, straight, compliant OA composed of the intracranial (IOA), optic canal (OC), and extracranial (EOA) segments. The lengths of the IOA, OC, and EOA segments used were those most commonly found in humans (Table 3).

**Table 3:** Geometrical parameters of the ophthalmic artery.

OA wall parameters	Value (mm)
initial lumen diameter	1.3
length of the OA ^39^	25.783
length of (IOA) ^39^	4.116
length of (OC) ^40^	12
length of (EOA) ^39^	9.667
wall thickness ^41^	0.177

*OA* the ophthalmic artery, *IOA* intracranial segment of the ophthalmic artery, *EOA* extracranial segment of the ophthalmic artery, *OC* optic canal.

The OA lumen volume and wall thickness were assumed to be unaffected by aging or any other processes (Table 3).

The material properties of the blood and OA wall corresponded to those of an average healthy human (Table 4).

**Table 4:** Basic parameters of blood and wall of the ophthalmic artery.

	Value	Units
**Blood properties**		
Effective dynamic viscosity ^42^	0.003675	Pa·s
Density ^43^	1060	kg·m^−3^
**OA wall properties**		
Density ^44^	1100	kg·m^−3^

The blood was modeled as an incompressible, homogenous, Newtonian fluid^34^. From basic theoretical considerations, the Reynolds number in the OA was calculated as less than 150, while the Womersley number was less than 1, which correspond to the prescribed laminar, fully developed velocity profile at the inlet. The magnitude of the blood flow velocity changed over time according to the typical OA velocity waveform (Fig. 7). To the best of our knowledge, only the peak ophthalmic arterial blood pressure values are available^35^. Therefore, the ophthalmic arterial blood pressure waveform was prescribed at the outlet as a rescaled version of the velocity waveform with the peak pressure values corresponding to those of a healthy human (Table 5).

**Table 5:** Basic simulation parameters.

Simulation parameters	Value	Units
Duration of one heartbeat pulse cycle ^45^	1	s
Systolic arterial blood pressure ^35^	80	mmHg
Diastolic arterial blood pressure ^35^	40	mmHg

To capture the exponential stiffening and the anisotropy, the OA walls were modelled as a fiber-reinforced, hyperelastic, nearly incompressible material with two mechanically equivalent fiber families^36^. To the best of our knowledge, the values of the additional artery wall parameters, required by the constitutive material model for the OA wall are unavailable, so internal carotid artery parameters were used instead (Table 6). The mean fiber directions of the two fiber families were defined according to a previously proposed method^29^.

**Table 6:** Ophthalmic artery wall parameters used for fiber-reinforced double layer model based on internal carotid artery wall parameters according to ^46^.

Arterial wall parameters	Value
Isotropic, kPa	29.7
Anisotropic, kPa	27.8
Anisotropic,	64.2
Fiber angle, deg	22.0
Dispersion,	0.8

The IOA segment was compressed by the ICP. No additional pressure acted on the OC segment, and the EOA segment was compressed by the intraorbital pressure (Pio) and the added external pressure (Pe). It was assumed that ICP, Pio, and Pe were uniformly distributed on the outer surface of the artery wall. To limit the rigid artery wall motion, a spring foundation with a relatively small a spring constant was prescribed on the interface boundary such that would not alter the stress field.

The geometry was discretized into 71,644 finite elements with 49,734 linear elements for the blood domain and the remaining 21,910 quadratic elements for the artery wall domain.

The fluid–structure interaction (FSI) model considering the two-way coupling between blood and artery wall was solved with COMSOL Multiphysics software (v.5.1. COMSOL AB, Stockholm, Sweden). The Navier–Stokes equations in the Arbitrary Lagrangian Eulerian (ALE) formulation were used to describe the dynamics of the blood flow, while the equation of motion in the material configuration was used to describe the dynamics of the artery walls. The computational mesh of the blood domain was moved according to the Winslow smoothing method^37,38^. A linear MUltifrontal Massively Parallel Sparse direct Solver (MUMPS) was used in conjunction with a nonlinear Newton method. For time stepping, the variable-order-accuracy (from first to second) Backward Differentiation Formula (BDF) solver was used with an adaptive time stepping routine. A relative global tolerance of $10^{ - 3}$ and a scaled global absolute tolerance of 5 × 10^−4^ were used for all solved variables (Table 7).

**Table 7:** Locations of cross-sections were data was extracted.

Simulation parameters	Value (mm)
distance from the OA starting location at which the data was collected at IOA segment	2.058
distance from the OA starting location at which the data was collected at EOA segment	20.834

*OA* the ophthalmic artery, *IOA* intracranial segment of the ophthalmic artery, *EOA* extracranial segment of the ophthalmic artery.

## Appendix 2: Definition of blood flow factor used for non-invasive ICP monitoring

The variations of the blood flow velocity parameters and OA cross-sectional area in the IOA and EOA can be compared by the integral factors derived from the intensity of the reflected ultrasound Doppler signal. The blood flow factor ($BFF$) is defined as an integral of the difference of logarithms of Doppler signal intensity *P*_*s*_ obtained from the IOA and EOA:

1 $$BFF\left( t \right){{ = = ~}}\mathop \smallint \limits_{{\uptau {{ = 0}}}}^{{{{10}}}} \mathop \smallint \limits_{{{u}}} \left| {{{~ln}}\left( {\frac{{P_{{su,IOA}} {{(t~{-}~}}\uptau {{,u)}}}}{{P_{{su,EOA}} {{(t~{-}~}}\uptau {{,u)}}}}} \right)} \right|{{du}}\;{{d}}\uptau {{~,~t~ = ~[10,~20,}} \ldots {{,~3590]~s,}}$$

where *P*_*su*_(*t*, *u*)—is the ultrasound signal intensity distribution over the velocity *u* measured by the Doppler signal. In order to compare $BFF$ with the ICP monitored non-invasively, the factors were processed. The factor $BFF$ and invasively monitored *ICP*_*inv*_ were smoothed by a moving average with ${\text{T}}_{{{\text{smooth}}}} = 60$ s, and the smoothed factor $\overline{BFF}$ was rescaled according to $\overline{{{\text{ICP}}_{{{\text{inv}}}} }}$ values by:

2 $${\text{f(t) = a + b }}\cdot \overline{BFF} {\text{(t)}}{.}$$

The scale coefficients *a* and *b* must satisfy the condition of equality of standard deviations and the means of rescaled factor *f* and $\overline{{{\text{ICP}}_{{{\text{inv}}}} }}$:

3 $$\left\{ {\begin{array}{*{20}c} {{\text{std(f(t)) = std(}}\overline{{{\text{ICP}}_{{{\text{inv}}}} }} {\text{(t))}}} \\ {{\text{mean(f(t)) = mean(}}\overline{{{\text{ICP}}_{{{\text{inv}}}} }} {\text{(t))}}} \\ \end{array} ,t = \left[ {T_{smooth} ,{ }t_{end} } \right],} \right.$$
